# ASSOCIATION OF PHYSICAL ACTIVITY WITH NEUROFILAMENT LIGHT IN NONDEMENTIA OLDER ADULTS AND THE MEDIATION OF FRAILTY

**DOI:** 10.1093/geroni/igae098.1460

**Published:** 2024-12-31

**Authors:** Sofia Liu, Mengchi Li, Miranda McPhillips, Russell Calderon, Junxin Li

**Affiliations:** Johns Hopkins University School of Nursing, Baltimore, Maryland, United States; Xi’an Jiaotong University, Xi’an, Shaanxi, China (People’s Republic); Villanova University, Villanova, Pennsylvania, United States; Johns Hopkins University, Lehigh Acres, Florida, United States; Johns Hopkins School of Nursing, Baltimore, Maryland, United States

## Abstract

Neurofilament light (NfL) is a biomarker for neuronal health, indicative of neural damage and decline in older adults. Physical activity (PA) is known to promote healthy aging and prevent frailty. However, the interplay between PA, physical frailty, and NfL remains underexplored. This study examines the associations between PA and NfL and the mediating role of frailty using baseline data of 95 community-dwelling non-dementia older adults in a randomized controlled trial. PA was measured using ActiGraph Link and Physical Activity Scale for the Elderly (PASE). Frailty was assessed using the Fried frailty phenotype, and NfL levels were determined from plasma samples. Multiple linear regressions, adjusting for sociodemographic and health factors, tested the association, and Baron and Kenny’s approach was used to examine frailty’s mediation effect. Participants were aged 70.0 ± 6.0 years, 80% female, 28.6% Black, had 2.8 ± 1.9 chronic conditions, and 28% had mild cognitive impairment (MCI). In the adjusted models, a higher level of Actigraph-measured PA was associated with lower NfL (β= -7.4, 95% confidence interval (CI) = [-13.4 to -1.3]). The frail group showed increased NfL compared to the robust (β = 26.3, 95% CI = [11.8 to 40.8]) and pre-frail (β = 25.5, 95% CI = [39.9 to 11.1]) groups. Further, frailty status significantly mediated the PA-NfL relationship. In summary, increased PA level was associated with reduced NfL, and frailty status mediated the associations in our sample of older adults with normal cognition or MCI. PA interventions may reduce frailty-related neuronal damage; further study is needed.

